# Hazardous drinking among young adults seeking outpatient mental health services

**DOI:** 10.1186/s13722-016-0060-y

**Published:** 2016-08-09

**Authors:** Anna E. Ordóñez, Rachel Ranney, Maxine Schwartz, Carol A. Mathews, Derek D. Satre

**Affiliations:** 1 Department of Psychiatry and UCSF Weill Institute for Neurosciences, University of California, 401 Parnassus Avenue, San Francisco, CA 94143 USA; 2 Division of Research, Kaiser Permanente Northern California Region, 2000 Broadway, 3rd Floor, Oakland, CA 94612 USA; 3 Office of Clinical Research, National Institute of Mental Health, 6001 Executive Blvd. MSC 9669, Bethesda, MD 20892 USA; 4 Department of Psychiatry, University of Florida, 100 S Newell Drive, Gainesville, FL 32610 USA

**Keywords:** Alcohol, Hazardous drinking, Cannabis, Depression, Mental health, Young adults

## Abstract

**Background:**

Alcohol use can have a significant negative impact on young adults in mental health treatment. This cross-sectional study examined prevalence and factors associated with hazardous drinking among young adults seeking outpatient mental health services, rate of alcohol use disorders (AUDs), and the relationship between hazardous drinking and other types of substance use.

**Methods:**

Participants were 487 young adults ages 18–25 who completed self-administered computerized screening questions for alcohol and drug use. Alcohol use patterns were assessed and predictors of hazardous drinking (≥5 drinks on one or more occasions in the past year) were identified using logistic regression.

**Results:**

Of the 487 participants, 79.8 % endorsed prior-year alcohol use, 52.3 % reported one or more episodes of hazardous drinking in the prior year and 8.2 % were diagnosed with an AUD. Rates of recent and lifetime alcohol, tobacco and marijuana use were significantly greater in those with prior-year hazardous drinking. In logistic regression, prior-year hazardous drinking was associated with lifetime marijuana use (OR 3.30, p < 0.001; 95 % CI 2.05, 5.28), lifetime tobacco use (OR 1.88, p = 0.004; 95 % CI 1.22, 2.90) and older age (OR 1.18 per year, p < 0.001; 95 % CI 1.08, 1.29).

**Conclusions:**

In an outpatient mental health setting, high rates of hazardous drinking were identified, and drinking was associated with history of other substance use. Results highlight patient characteristics associated with hazardous drinking that mental health providers should be aware of in treating young adults, especially older age and greater use of tobacco and marijuana.

## Background

Substance use disorders, hazardous drinking and mental illness all peak in prevalence in early adulthood, yet few young adults receive appropriate services. For example, the 2011 National Household Survey on Drug Use and Health (NHSDUH) found that the 1-year prevalence of illicit drug or alcohol abuse or dependence increased from 7 % among 12–17 year olds to 19 % for 18–25 year olds, decreasing to 6 % for individuals over 25 [1]. The same report found that adults ages 18–25 had higher rates of mental illness and were less likely to receive treatment in the prior year than older adults.

Alcohol use can adversely impact symptom severity and treatment of co-occurring mental illness [2–5]. Reduced response to antidepressants and increased risk of side effects have been reported with even moderate levels of alcohol use [5]. In the STAR*D depression treatment cohort, individuals with major depressive disorder and co-occurring substance use disorders (including alcohol) had earlier onset of depression, greater severity and functional impairment, and higher rates of suicide attempts and completed suicide [3]. Similarly, while many individuals with anxiety disorders use alcohol for short-term symptom relief, drinking can ultimately make anxiety more severe [2, 4]. These associations highlight the need to assess alcohol and drug use patterns among young adults with mental health problems, in order to understand potential symptom exacerbation and medication interaction risks. Assessment could also help to identify which individuals may benefit from psychiatry-based brief interventions to reduce harmful drinking patterns, and who should be referred to specialty care addiction treatment.

Apart from the potential value of brief interventions, screening provides benchmark medical record data at intake to help providers track potential changes in drinking over time. Some studies suggest that screening alone could help to reduce drinking [6, 7]. In the clinician’s guide to identifying and treating drinking problems in health care settings, the National Institutes on Alcohol Abuse and Alcoholism (NIAAA) recommends asking how many times in the past year individuals have had 5 or more drinks for men and 4 or more for women [8]. In 2009 Smith et al., reported a sensitivity of 88 % and specificity 67 % of this cutoff in detecting a current (AUD) in a primary care setting [9]. Using the Alcohol Use Disorders Identification Test (AUDIT) as reference, Massey et al. [10] reported 96 % sensitivity and 82 % specificity of screening question to detect harmful drinking in an alert nonpsychotic consult-liaison population. In the present study we used a similar cutoff (5+ drinks for both men and women) drawn from electronic health record intake data in a psychiatry clinic setting to examine prevalence and correlates of hazardous drinking in young adults. The same cutoff was used for both sexes as this was the information available from the data, which was based on a graduated frequency measure that did not adjust quantities based on sex.

Although young adults are at high risk for alcohol-related problems [11], studies evaluating drinking patterns and their association with clinical characteristics are lacking. This study evaluated self-reported alcohol use patterns and the association between prior-year hazardous drinking and potentially relevant patient characteristics, including gender, age, clinician-assigned psychiatric diagnosis, and other substance use in a sample of young adults presenting for initial mental health treatment. We hypothesized that prior-year hazardous drinking would be associated with an AUD diagnosis, with other common psychiatric diagnoses, in particular, anxiety and depression, and with other types of substance use prevalent in this population such as tobacco and marijuana.

## Methods

### Participants and measures

Study participants were adults ages 18–25 seeking psychiatric services in an outpatient clinic in a university medical center. This clinic provides a range of assessment and treatment services, including medication management and individual and group psychotherapy. The clinic has no formal services for patients primarily seeking alcohol or drug treatment. Individuals seeking such services are pre-screened by telephone by clinic staff and referred to local specialty care programs.

The sample included all individuals who presented to the clinic for initial evaluation between September 14th, 2005 and June 29th, 2011, were between the ages of 18 and 25 at intake, and completed routine computerized questionnaires, including a self-administered Electronic Health Inventory (EHI) [12], Beck Depression Inventory-II (BDI-II) and a clinical interview. Other than age range and intake dates, there were no exclusion criteria.

The EHI was completed on private computers in the clinic waiting area. It included questions about demographic characteristics, current and past medical history, and patterns of substance use for alcohol, cannabis and tobacco. For each substance, participants were asked if they had ever used that substance during their lifetime. Positive responses prompted questions on duration and frequently of use. Providers received a printed copy of the EHI questionnaire results for use in evaluation of new patients at intake. The University of California, San Francisco Committee on Human Research approved the study, including the examination of de-identified records of patients who had an initial clinic visit during the study time period.

Participants who endorsed any lifetime alcohol or cannabis/marijuana use were asked the timing of most recent use (in years, months or days) prior to intake. Alcohol use questions included usual quantity consumed per occasion (in standard drinks), frequency of use in the past 30 days and number of days in the past year when 1–2, 3–4, 5–7, and ≥8 drinks were consumed on one occasion (graduated frequency method) [13]. Combining the responses of any consumption of 5–7 or ≥8 drinks consumed on one occasion in the past year, hazardous drinking was defined for this analysis as any past-year consumption of 5 or more drinks on one occasion, consistent with the definition used by the NHIS (5+ drinks for both women and men) during the same time period [14]. While NIAAA currently recommends a different cut-off for hazardous drinking in men (5+) and women (4+), data were not available to assess this distinction.

### Substance use and psychiatric disorder diagnoses

By chart review, we obtained all assigned Diagnostic and Statistical Manual of Mental Disorder, Fourth Edition Text Revision (DSM-IV-TR) [15] diagnoses listed on each participants’ standardized initial intake evaluation form, as assigned and documented by the clinician. Blinded to responses on the EHI, a study research assistant reviewed and coded all listed diagnoses. We coded only definite diagnoses, excluding “rule out” diagnoses.

We coded drug use disorder positive if abuse or dependence was diagnosed for the following drugs: amphetamine, cannabis, opiates, methamphetamine, mushrooms, benzodiazepines, cocaine, stimulants, or if polysubstance abuse was diagnosed. Given the young age of the sample, disorders in remission would still be temporally relatively recent. Therefore, no distinction was made between diagnoses in remission or active. We coded alcohol use disorder (AUD) positive if alcohol abuse or dependence was diagnosed. Likewise, no distinction was made between diagnoses in remission or still active. We coded depressive disorder positive if major depressive disorder, dysthymia, or depression not otherwise specified (NOS) was diagnosed. Similarly, we coded an anxiety disorder if anxiety disorder NOS, generalized anxiety disorder, social anxiety disorder, panic disorder, specific phobia, post-traumatic stress disorder or obsessive compulsive disorder was diagnosed. We coded bipolar disorder positive if bipolar affective disorder (BAD) type I, II or NOS was diagnosed. We coded psychotic disorder positive if schizophrenia, schizoaffective disorder, delusional disorder or psychosis NOS was diagnosed. We coded attention deficit hyperactivity disorder (ADHD) positive if ADHD (inattentive, hyperactive, or combined type) or ADHD NOS was diagnosed. We coded eating disorder positive if anorexia, bulimia or eating disorder NOS was diagnosed.

### Analyses

We linked self-reported demographic and substance use data from the EHI to diagnostic data from the chart review to create a single dataset for analysis. We compared differences in alcohol use rates between men and women using the χ^2^ test, and differences in BDI-II score and mean quantity of alcohol consumed between women and men using t tests. Similarly, using χ^2^ tests for categorical variables and t tests for continuous variable, rates of alcohol, tobacco and marijuana use, as well as rates of specific psychiatric diagnoses at intake were examined by prior-year hazardous drinking. Underage alcohol use was also examined (rate of any hazardous drinking among those ages 18–20 vs. 21–25). Given that participants could be assigned several diagnoses at intake, individual diagnoses were not included in regression models (as they were not independent of each other). Instead, we assessed diagnostic burden as indicated by the number of diagnoses assigned at intake. We used a single logistic regression model to test the association between number of psychiatric diagnoses, any lifetime use of tobacco and cannabis, age, race/ethnicity and gender as potential predictors of participants reporting any hazardous drinking in the prior year. We used STATA version 13 for all analyses.

## Results

During the study intake period, 487 new patients between the ages of 18–25 years were admitted. The sample was racially diverse and predominantly female, and included a substantial percentage of students (Table 1).

**Table 1:** Demographic characteristics and occupational status of adults ages 18–25 seeking outpatient mental health treatment (N = 487)

Variables	Mean (SD) or %
Gender (%)	
Men	33.9
Women	66.1
Age (mean, SD)	22.2 (±2.3)
Men	21.9 (±2.4)
Women	22.3 (±2.3)
Race (%)	
Asian	15.2
Black	2.5
White	62.6
Other	19.7
Hispanic origin (%)	9.9
Education (%)	
High school grade 7–12	4.6
High school graduate or GED	47.3
Completed technical training	3.2
College graduate	27.8
In or completed graduate training	17.1
Occupational status (%)	
Student	44.8
Employed (full or part-time)	31.7
Unemployed	21.5
Disability	2.0

Lifetime alcohol use was endorsed by 85.4 % of the sample, prior year alcohol use was endorsed by 79.8 %, and 52.3 % reported prior year hazardous drinking. Frequency of hazardous drinking was: 1–5 times a year (22.8 %), 6–11 times a year (11.5 %), about once a month (3.3 %), 2 or 3 times a month (7.0 %), once or twice a week (6.2 %), 3 or 4 times a week (0.8 %), nearly every day (0.5 %). Lifetime marijuana use was endorsed by 66.7 % of participants and prior year marijuana use was endorsed by 48.1 % of the sample. There were no significant gender differences in any of the above rates. AUD diagnoses were present in 8.2 % of the sample, and were twice as prevalent among women (10.3 %) compared to men (4.2 %) (χ^2^ = 5.2, p = 0.02). Participants over age 21 (N = 313) endorsed significantly greater prior-year hazardous drinking than those under 21 (N = 174) (58.8 % vs. 41.2 % respectively; χ^2^ = 14.5, p < 0.001) (not shown).

The most prevalent psychiatric diagnoses at intake were depression (55 %) and anxiety disorders (43.3 %) with no gender differences. BDI-II scores were available for 395 participants. Overall mean BDI-II score was 22.2 (SD = 10.8), indicative of moderate depression, with a significant difference between men (mean = 18.6, SD = 10.8) and women (mean = 24.1, SD = 13.4) (p ≤ 0.001) (not shown).

Overall, rates and frequency of alcohol, tobacco and marijuana use were significantly greater in those who endorsed hazardous drinking in the prior 12 months compared to those who didn’t (Table 2). Rates of AUDs were four times greater among those who endorsed hazardous drinking in the prior 12 months, compared to those who didn’t (Table 2). Rate of psychotic disorders among those who endorsed prior 12-month hazardous drinking were less frequent compared to those who denied hazardous drinking in the prior 12 months. There were no significant differences in the rates of other psychiatric disorders among those who did and those who did not endorse hazardous drinking in the prior 12 months.

**Table 2:** Substance use patterns and psychiatric diagnoses of young adults ages 18–25 seeking outpatient mental health treatment by hazardous drinking status

Variable	Hazardous drinking in the prior 12 months Total N = 487				
	No hazardous drinking N = 232 (47.6 %)		≥1 days of hazardous drinking N = 255 (52.3 %)		p value
	Mean or %	SD	Mean or %	SD	
Alcohol use (%)					
Lifetime	*69.4*		*100*		<*0.0001*
Prior year	*59.1*		*98.8*		<*0.0001*
Prior month	*37.9*		*87.8*		<*0.0001*
Usual quantity of drinks consumed per occasion	*0.9*	±1.1	*3.1*	±1.9	<*0.0001*
Number of days alcohol was consumed in prior 30 days	*2.2*	±4.5	*6.4*	±6.5	<*0.0001*
Tobacco use (%)					
Lifetime	*38.8*		*65.9*		<*0.0001*
Prior year	*22.4*		*51.0*		<*0.0001*
Prior month	*21.1*		*38.8*		<*0.0001*
Marijuana use (%)					
Lifetime	*49.6*		*82.4*		<*0.0001*
Prior year	*29.7*		*64.7*		<*0.0001*
Prior month	*16.0*		*40.0*		<*0.0001*
Psychiatric diagnoses at initial intake (%)					
Depressive disorder	53.9		56.1		0.6261
Anxiety disorder	42.2		44.3		0.6448
Bipolar disorder	11.6		17.3		0.0794
Eating disorders	14.2		11.8		0.4193
Psychotic disorder	*16.4*		*8.24*		*0.0059*
Drug use disorder (excluding tobacco and alcohol)	5.17		9.41		0.0741
Alcohol use disorder	*3.02*		*12.9*		*0.0001*
Attention deficit disorder	7.33		6.67		0.7750
PTSD	6.47		4.31		0.2914
Mean number of diagnoses at initial intake	*2.02*	±*1.1*	*2.36*	±*1.3*	*0.0300*

Significant differences appear in italics

Logistic regression analysis was used to examine predictors of prior-year hazardous drinking (Table 3). The single model included number of diagnoses at intake, age, gender, race, and any lifetime marijuana or tobacco use. Variables positively associated with prior-year hazardous drinking included lifetime marijuana use (OR 3.30, p < 0.001; 95 % CI 2.05, 5.28), lifetime tobacco use (OR 1.88, p = 0.004; 95 % CI 1.22, 2.90) and older age (OR 1.18 per year, p < 0.001; 95 % CI 1.08; 1.29) (Table 3). Results from sensitivity analyses using prior-year cannabis and smoking measures (which occurred during the same time frame as the hazardous drinking) were similar, and the significance of the measures in predicting prior-year hazardous drinking did not change (not shown).

**Table 3:** Factors associated with prior-year hazardous drinking in young adults seeking outpatient psychiatric services (N = 487)

Predictor	OR	p value	95 % CI
Number of psychiatric diagnoses at intake	1.06	0.459	0.90–1.25
Lifetime cannabis use	*3.30*	*<0.001*	*2.05*–*5.28*
Lifetime tobacco use	*1.88*	*0.004*	*1.22*–*2.90*
Age in years	*1.18*	*<0.001*	*1.08*–*1.29*
Female gender	0.98	0.932	0.64–1.50
Race (reference: white)			
Black	0.52	0.372	0.13–2.17
Asian	*0.43*	*0.007*	*0.23*–*0.80*
Other	1.20	0.470	0.73–2.00

Results are from a single multivariate logistic regression

Significant differences appear in italics

## Discussion

This study examined the relationship of prior-year hazardous drinking to patterns of alcohol, tobacco and cannabis use, as well as psychiatry diagnoses, in a young adult outpatient psychiatry sample. In this treatment-seeking sample, cannabis and tobacco use as well as older age were significant predictors of hazardous drinking.

These results highlight the high rates of hazardous drinking in a young adult population seeking mental health treatment, and the need for systematic screening in this group. The levels of prior-year hazardous drinking in our study were approximately twice as high in men (53.3 vs. 23.7 %) and five times higher in women (51.9 vs. 10.3 %) than in individuals of the same age, during the same time frame in the National Health Interview Survey (NHIS) [14]. Our sample also included a substantial proportion of students, we found comparable rates to those seen in college students (approximately 45 % prior-month hazardous drinking) [16]. These rates are also higher than those seen in a study of hazardous drinking among adults with moderate or greater depression symptoms from the same clinic setting [17]. This sample (N = 1183) ranged in age from 18 to 91, with a mean age of 42.2 (SD = 14.7) 47.5 % of men and 32.5 % of women reported prior-year hazardous drinking, compared to 53.3 and 51.9 % for men and women in our young adult sample. In addition, younger age was an independent predictor of hazardous drinking in this larger clinic sample. Thus, the clinical setting, student composition and age of participants may help to explain our findings.

Based on this same previous study in the psychiatry clinic with a mean age of 42.2 [16], the most common primary psychiatric diagnosis assigned to patients following their first visit was major depressive disorder (48.4 %), followed by bipolar disorder (14.8 %), anxiety disorders (11.2 %), depressive disorder not otherwise specified (7.5 %), mood disorder not otherwise specified (4.3 %), adjustment disorders (4.3 %), schizophrenia (1.2 %), and 9.1 % all other diagnoses combined. For most diagnoses, rates were similar to those in the current sample. The exception is anxiety diagnoses, which were identified at a higher rate in the current sample. A potential explanation for the difference is sample selection (participants scored 10+ on the BDI-II) as well as the way in which diagnoses were identified. The prior study measured only primary diagnoses, while the current young adult study used manual chart review to include all diagnoses assigned by providers.

Although rate of AUD assignment was low relative to the rate of prior-year hazardous drinking, especially among men, any self-reported prior-year hazardous drinking was associated with a fourfold higher rate of an AUD diagnosis. These findings highlight the relevance of hazardous drinking screening among young adults seeking mental health treatment as a component of psychiatric evaluation and treatment [2, 3, 5] and an indicator of a possible AUD. It is noteworthy that in our sample AUD diagnoses were twice as prevalent in woman as in men. This is in contrast to many prior epidemiological studies, including the National Epidemiologic Survey on Alcohol and Related Conditions (NESARC) [18], which found rates of alcohol abuse and dependence to be a little more than double in men compared to women. It is possible that, rather than reflecting actual gender differences in AUD diagnoses, the higher rate of AUD among women in our sample reflects a greater concern from providers regarding problematic drinking in treatment seeking young women than in young men. This pattern has previously been described in a study of the Veterans Health Administration, which found race and gender differences among VA patients with clinically recognized AUDs [19]. That study outlines the importance of validating diagnoses against structured gold-standard clinical assessments to better understand whether providers are over or under-identifying AUDs.

Analyses of the relationship of psychiatric diagnoses to hazardous drinking indicated that, aside from AUDs, no single psychiatric disorder was particularly associated with increased rates of hazardous drinking. The finding of hazardous drinking being associated with lower rates of psychotic disorder diagnosis was not anticipated. There is a large body of data regarding comorbid substance abuse and psychosis. For example, psychosis has been associated with frequent cannabis use in national surveys [20]. Similarly, several studies have described comorbid AUDs in patients with psychosis [21, 22]. It is therefore unexpected that among all diagnoses, hazardous drinking would be negatively associated with psychotic disorder. One possible explanation is that, given that all participants were seeking outpatient mental health treatment, the variability in the sample was more limited than in those seen in other studies.

Study findings have important implications for clinical practice. Screening for hazardous drinking should be conducted with all psychiatric patients, regardless of diagnosis. The finding that any marijuana use was associated with 3.3 fold greater odds of prior-year hazardous drinking is also noteworthy. This finding was consistent with previous literature in college students, which found that those who use both marijuana and alcohol are more likely to experience alcohol and other drug problems, including higher mean number of drinks per occasion [23]. Helping clinicians be aware of the frequency of co-occurring marijuana use with hazardous drinking may represent an additional opportunity to improve identification of alcohol and other drug problems.

Mental health clinics are important settings in which to address hazardous drinking and identify AUDs. Individuals with AUDs are more likely to seek care in mental health settings than in specialized addiction treatment programs [24]. While effective interventions to reduce co-occurring alcohol problems exist, providers in psychiatry clinics often fail to identify warning signs of problematic drinking and overlook opportunities to intervene [25, 26]. Interventions such as motivational interviewing could be important supplements to mental health treatment [27–29]. The Screening, Brief Intervention and Referral to Treatment (SBIRT) model promoted by the U.S. Substance Abuse and Mental Health Services Administration (SAMHSA) [30] is another example of a potential supplement to existing mental health care. SBIRT is a public health-based approach to early intervention for at-risk individuals identified in primary care and other health settings. Implementing these interventions in general psychiatric treatment for young adults, as well as identifying and referring AUDs to specialty addiction treatment when indicated, could help reduce hazardous drinking and improve overall patient care.

## Limitations

This study had several limitations. While computerized self-report measures are valid, under-reporting of alcohol and cannabis use by patients would make our prevalence rates conservative. In addition, the clinic that served as the study site routinely referred patients primarily seeking care for alcohol and drug problems to specialty care treatment programs, which may also lead to lower prevalence rates of hazardous drinking in our population compared to some other psychiatric service settings. Similarly, our study used provider-assigned diagnoses and did not systematically assess AUDs using structured interviews, resulting in potential under- and/or over-estimate of AUD rates and hindering our ability to determine the sensitivity and specificity of prior-year hazardous drinking as a predictor of AUDs. In addition, the lack of distinction between active AUD or substance use disorder diagnoses and those in remission limits the correlation of hazardous drinking in our study to any lifetime AUD or substance use disorder diagnoses. However, the low number of clinician-assigned AUDs in the context of hazardous drinking remains noteworthy. Lastly, using a lower cutoff for hazardous drinking for women than for men (4 drinks per occasion rather than 5), would increase sensitivity of “at risk” drinking in this group, and is often used in population-based and clinical studies. The use of a higher cutoff in this study may make our estimates of hazardous drinking among women conservative.

## Conclusions

This study examined the extent of hazardous drinking, alcohol and other substance use patterns, provider-assigned AUDs, and co-occurring psychiatric disorders among young adults in an outpatient psychiatry clinic. Prior-year hazardous drinking rates for both men and women were substantially higher than those found in studies of young adults in the general population. Lifetime marijuana use and tobacco use significantly predicted prior-year hazardous drinking. There was a strong association of prior-year hazardous drinking with a clinician-assigned AUD, even though overall rate of AUD diagnosis was relatively low. Outpatient mental health service settings offer an excellent opportunity for early identification and intervention to reduce alcohol and other substance use among young adults.
